# Comparison of a palm-based biometric solution with a name-based identification system in rural Bangladesh

**DOI:** 10.1080/16549716.2022.2045769

**Published:** 2022-03-28

**Authors:** Fatema Khatun, Rebecca Distler, Monjur Rahman, Brian O’Donnell, Noni Gachuhi, Manoj Alwani, Yang Wang, Anisur Rahman, J Frederik Frøen, Ingrid K Friberg

**Affiliations:** a Norwegian Institute of Public Health, Oslo, Norway; b International Centre for Diarrhoeal Disease Research, Bangladesh (icddr,b), Dhaka, Bangladesh; c Element Inc, New York, NY, USA; d Intellectual Ventures, Global Good Fund, Bellevue, WA, USA; e University of Bergen, Bergen, Norway; f Tacoma-Pierce County Health Department, Tacoma, WA, USA

**Keywords:** Biometrics, Bangladesh, palmprint, biometric identification, DHIS2

## Abstract

**Background:**

Unique identifiers are not universal in low- and middle-income countries. Biometric solutions have the potential to augment existing name-based searches used for identification in these settings. This paper describes a comparison of the searching accuracy of a palm-based biometric solution with a name-based database.

**Objective:**

To compare the identification of individuals between a palm-based biometric solution to a name-based District Health Information Software 2 (DHIS2) Android application, in a low-resource setting.

**Methods:**

The study was conducted in Chandpur district, Bangladesh. Trained data collectors enrolled 150 women of reproductive age into two android applications – i) a name-based DHIS2 application, and ii) a palm-based biometric solution – both run on tablets. One week after enrollment, a different research team member attempted to re-identify each enrolled woman using both systems. A single image or text-based name was used for searching at the time of re-identification. We interviewed data collectors at the end of the study.

**Results:**

Significantly more women were successfully identified on the first attempt with a palm-based biometric application (84%) compared with the name-based DHIS2 application (61%). The proportion of identifications that required three or more attempts was similar between name-based (7%, CI 3.7–12.3) and palm-based biometric system (5%, CI: 1.9–9.4). However, the total number of attempts needed was significantly lower with the palm-based solution (mean 1.2 vs. 1.5, p < 0.001). In a group discussion, data collectors reported that the palm-based biometric identification system was both accurate and easy to use.

**Conclusion:**

A palm-based biometric identification system on mobile devices was found to be an easy-to-use and accurate technology for the unique identification of individuals compared to an existing name-based application. Our findings imply that palm-based biometrics on mobile devices may be the next step in establishing unique identifiers in remote and rural settings where they are currently absent.

## Background

Low- and middle-income countries struggle with the lack of unique personal identifiers for their populations [1]. The health system is additionally burdened by this gap as the ability to correctly identify an individual is critical to providing quality care. Without correct identification, continuity of care along longitudinal programs, such as pregnancy care, and across the health system, is challenging, with invisible gaps in information and knowledge for patients and providers. Even in high resource settings with universal personal identifiers, such as national personal identification numbers, identification errors are common; up to 10% of people may be misidentified at some point during their interaction with the health system [2,3]. There is limited evidence around the level of misidentification in rural low- and middle-income countries, but it is expected to be considerably high. Consequences can range from treatment errors, therapeutic failures, difficulties in following-up a patient, wastage of healthcare resources and even death [4]. Fatal outcomes may occur when medication is not delivered to the critically ill patient or is delivered to the allergic patient. In Bangladesh, where there is a lack of an established unique identifier for individual records, the healthcare provider may fail to accurately identify, and follow-up missed antepartum care (ANC) visits for pregnant women. This could potentially lead to adverse pregnancy outcomes and higher maternal and neonatal deaths [4].

Individual-level electronic health records can bridge data quantity and timeliness gaps, but only if the provider can correctly link the individual with their corresponding record. For example, knowing a pregnant woman’s medical and personal history helps to appropriately and adequately, tailor the healthcare choices and services being offered to her. However, without unique identifiers, the woman could be entered multiple times in one system, resulting in poor quality summary data and potentially poor quality of care because health information from previous visits or other providers is not available to the current provider. Unique identifiers are also important in facilitating the sharing of records across multiple providers, and reducing waste through over-provision of services [5].

Several unique identification systems exist which have the potential to improve the recognition of individuals. They include unique personal numbers, identity cards or barcodes. However, such methods require an established uniqueness of individuals in the first place, and have the potential be lost or misplaced, and thus be underutilized [6]. Biometric recognition, such as of fingerprints, irises, voices or faces, are possible alternatives. In many different low- and middle-income countries, the use of fingerprint biometrics in healthcare has proven feasible and cost-effective [7]. In Benin, a biometric-based vaccination registry (VaxTrac) uses the child’s fingerprint to keep track of the vaccine doses provided to each child to avoid both missed doses and double doses [8]. In South Africa and Malawi, fingerprint-enabled logs of anti-retroviral therapies support the tracking of treatment adherence for HIV-positive patients [9]. In Kenya and Ghana, fingerprints have been used to establish individual identity in health and demographic surveillance [9]. In India, Operation ASHA runs a tuberculosis treatment program where patients are recorded using a fingerprint-linked text messaging system [10,11]. However, the above studies were conducted in health facilities. To our knowledge, no published study has to date evaluated the use of any biometric method by community health workers for the identification of clients during household visits.

Despite the widespread use of fingerprint biometrics in healthcare, many limitations exist in low- and middle-income countries, including the documented poor specificity of fingerprints and limited internet connectivity to centralized databases in rural areas [12]. Typical biometric technologies utilize contact-based methods, such as the collection of finger or palm impressions or images, which raise hygiene concerns in public health contexts [13,14]. Moreover, in such settings, many people are involved in manual labour and their fingertips are often worn down, burnt or scarred. Compared to the finger pad, the palm offers a wider surface with more distinguishing features to better establish the uniqueness of an identity [15,16]. Most importantly, images can be captured from a distance utilizing a touchless technique, presenting a significant advantage in the context of the COVID-19 pandemic [17].

While there has been a shift toward contact-less methods globally, these techniques generally require an infrared scanner, a high-resolution camera, or other specialized hardware that is not always practical to implement in low resource settings [18,19]. To the best of our knowledge, no biometric system has been designed specifically for sustainable use in the developing world or delivered on cheap mobile devices. Touch-free palm-based biometric recognition is a novel concept in developing countries, which is why the majority of existing studies on this topic pertain to high-resource settings [20]. Furthermore, there is a need for a suitable biometric tool that primary health care providers can use during household visits to register clients for care, including maternal, child health, and family planning services. Element, Inc [21]. developed a palm-based biometric system, which uses the built-in camera on mobile devices to image palmprints and generate unique identifiers. The application runs on existing mobile devices and has the ability to work both online and offline, making it feasible in areas with less robust and reliable connectivity.

In this study, we compared a palm-based biometric android application for identification of individuals to a name-based District Health Information Software 2 (DHIS2) application, in a low-resource setting [22]. These findings can be used to improve the process of patient identification within the rapidly changing and digitizing health information systems in rural Bangladesh and other developing countries.

## Methods

### Study setting, population eligibility and design

In this pilot study, we registered women using two different mobile registration systems. During a follow-up visit after seven days, we searched for them within the same systems to quantify the speed, accuracy and ease-of-use of both systems in re-identifying women among a rural population in Bangladesh.

This study was conducted in Matlab North, a sub-district under Chandpur district. The study area is located 55 km away from the capital city of Dhaka, Bangladesh. This pilot study was carried out as part of an ongoing electronic health registry intervention trial in Matlab (eRegMat: ISRCTN69491836) to determine whether the palm-based biometric application should be included in the eRegMat trial to assist in the identification of women enrolled in the trial.

Women were eligible for participation in this pilot study if they were of reproductive age (15–45 years) and lived in three villages purposively selected for ease of access. Women were excluded if they were not likely to be available for reassessment one week after enrolment or if they had Mehendi (henna/ temporary tattoos using herbal dyes commonly used during religious occasions) on their hands. As Mehendi was not common on the hands used to develop the palm methodology, we chose to exclude them during this pilot study. A total of 150 women were enrolled, 50 per village, in April-May of 2018.

### Identification systems

#### Name-based registration system

**Figure 1.**
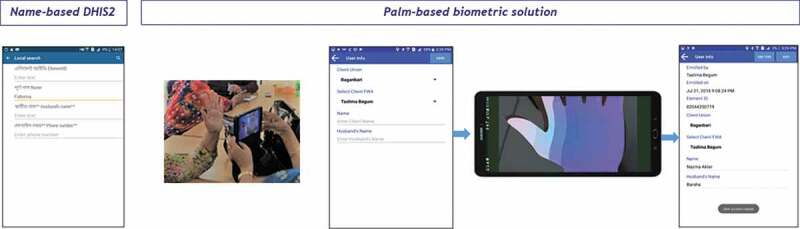
Name-based DHIS2 application and palm-based biometric solution enrolment procedure.

DHIS2 is a free and open-source web-based health management information system (HMIS) introduced by the University of Oslo and being used in over 74 low- and middle-income countries for collecting data, particularly for public health purposes such as facility reports and disease surveillance [23]. Bangladesh has adopted DHIS2 to allow collection, validation, analysis and visual representation of both individual and aggregated health data to inform public health policies and programs. Community health workers in the eRegMat trial collect data and provide care to participants using the DHIS2 Android application with standard search features and algorithms on mobile devices. Search is conducted offline, so mobile users only have access pre-downloaded records onto their devices before starting the search procedure. The DHIS2 search algorithm is both partial and combinatorial. If the user searches for a part of one woman’s name, results will include all records with any partial match of that name (e.g. ‘Far’ or ‘arh’ would match ‘Farhana’) (Figure 1). If the user searches for parts of a woman’s name *and* her study ID, results will include all records with a partial match for *both* search criteria. There is no so-called ‘fuzzy’ search to identify a ‘near match’ or common misspellings. There are no language restrictions on character type, meaning names may be entered and searched in either Bengali or English. However, names entered in Bengali cannot be found with English searching, and vice versa.

Since 2011, core developers at the University of Oslo have expanded DHIS2 features for capturing, analyzing, and sharing individual-level data. One result of this development has been the ‘eRegistry’, an app within DHIS2 specifically designed to integrate a dynamic search of patient records within the process for registering new clients and hiding records if a search yields too many results. However, we evaluated the standard Android setup as it is used in many DHIS2 implementations, including the eRegMat trial.

Within DHIS2, the study team created a registration form which included the following identifying characteristics for individuals: woman’s name, husband’s name, healthcare provider’s name, and study ID. Note that address identifiers were not included. Once on the search page in the Android app, the user must type in search criteria for at least one of these fields.

#### Palm-based biometric registration system

Element, Inc. has developed a deep learning-based solution for biometric identification. Deep learning is a branch of machine learning which has provided state of the art results in fields like computer vision, natural language processing, and security [21]. Element applies these techniques to biometric images of open palmprints captured by standard cameras on mobile devices, which allows the platform to run on existing low-end devices, rather than requiring additional, dedicated hardware.

Element’s palm-based identification system has two steps: enrolment and identification. For enrolment, several images of a person’s palm are captured (seven images of the open left palm in this implementation), which ensures clarity of images and captures variation in human behaviour. The deep learning model compresses these images into simpler representations (i.e. one dimensional vectors). The model was trained in such a way where images from the same person have similar vectors and images from different users have different vectors. The vectors from each enrolment image are combined to form a ‘user model’ which refers to the individual. This user model is a unique, highly abstract representation of the images which cannot be reverse engineered into the underlying photos. The seven images taken during enrolment are deleted automatically as soon as the user model is created and are not stored anywhere – a core security feature. The second step of the process is identification, in which the Element system takes one image of the palm to identify the person. In this step, the one-dimensional vector of a captured image is matched with the user model; if the vector is highly correlated, it returns a match. Once an image is captured, the Element system displays the result to the user (Figure 1).

**Figure 2.**
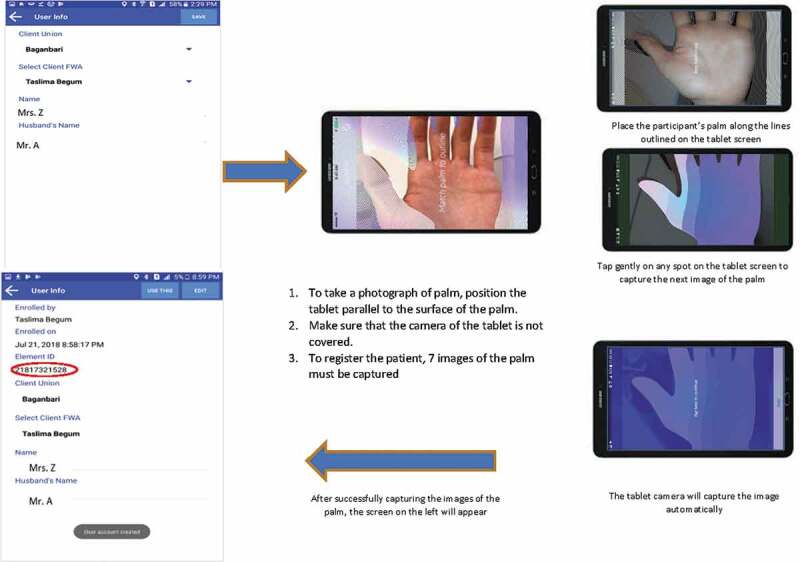
Registration and enrolment of participants with palm-based biometry.

For this trial, Element provided their system in a standalone palm-based biometric Android application (‘ePalm Matlab’) which was downloaded onto the Samsung Galaxy J-Max tablets procured for the eRegMat trial. A data entry form was created which included the woman’s name and husband’s name; union and health provider’s name were automatically assigned in the system when the user logged on (Figure 2). Address identifiers were not included.

Once the data entry form was filled out, the application prompted the data collector to capture seven images of the woman’s open left palm to create the user model. The application was designed so that enrolment could happen offline without internet connectivity. Once tablets gained connectivity, user models could sync across devices – enabling women to be enrolled on one device and identified on another.

### Training

Three female data collectors who had completed high school were given a two-day training in data collection using a Samsung tablet (Galaxy J Max SM-T285YD). The training covered registration in both mobile systems as well as using the systems to search for already enrolled women; successful completion of the training required passing a practice-based assessment in all aspects of data collection.

### Registration and searching

Each data collector obtained informed written consent from study participants at enrolment. Each enrolled woman was registered in both electronic systems during the same home visit, and then searched for during a re-identification home visit after 1 week by a different data collector on a different device. Following the initial registration of the woman in both electronic systems, she was given a card confirming her enrolment with a study identification number. Her name was not on the card. The data collector maintained a paper-based record of the enrolled women, including study enrolment ID and household identification information such as household number to facilitate finding the women during the re-identification phase of the trial (supplementary File 1).

At the end of enrolment, the data collectors synced the data in both systems using wi-fi and ensured that data for each enrolled woman was available in both systems for all data collectors. After one week, the data collectors were assigned to search for enrolled women in villages different from the villages where they had enrolled women. Each household location where an enrolled woman lived had been recorded on paper without any additional identifying features of the women. The data collector asked if anyone was enrolled in the trial at each known household. If yes, the data collector searched for the woman within the name-based database, using the woman’s name and her healthcare provider’s name. The healthcare provider’s name was used as an additional identifier to confirm and differentiate the identities of women with the same names or other matching details. After attempting to find this person with the name-based system, the data collector repeated the process with the palm-based biometric system. The data collector would search for the woman up to five times in both systems to identify women. The search was labelled as ‘not identified’ if study participants were not identified successfully within five attempts. A woman was considered correctly identified if the identification number in the electronic system matched the number on her study identification card. The data collector recorded on paper: 1) the success or failure of identification; 2) the number of attempts needed to correctly find the woman (up to a maximum of 5 attempts), and 3) a categorised perception of the time (<15 sec, 15–60 sec, more than 60 sec) needed to find the woman correctly. Perception was used rather than exact time to minimize the need for an additional observer during the study. No data from within the name-based DHIS2 system or the palm-based biometric system were used for analysis. The registrations were deleted after the trial was completed.

This project was approved by Regional Committees for Medical and Health Research Ethics of Norway (approval number REK sør-øst 2017/2468) and Ethical Review Committee of icddr,b (approval number PR-16054).

### Qualitative evaluation

A mini Focus Group Discussion (FGD) was conducted with the three data collectors by a researcher with a background in qualitative research. We choose a mini FGD to gather the collective voice of new technology usage in a rural area. Discussion topics included: 1) acceptance of imaging of the palms for biometric identification and perception of other biometric modalities (e.g, facial picture, fingerprint); 2) relative ease of use of the two identification applications; 3) relative reliability of the two applications; 4) challenges faced during study enrolment and re-identification, and 5) views on the potential utilization of either or both applications in the Matlab area. The discussion was recorded, transcribed, and translated. The results were manually synthesized.

### Data analysis

Data forms were re-checked by the research team for quality control and analyzed in STATA. Frequency distributions and 95% confidence intervals were calculated. The non-parametric Wilcoxon rank-signed test was used to evaluate the difference in the number of search attempts needed to identify a woman correctly.

## Results

A total of 150 women were enrolled in this trial. During the re-identification or search phase of the trial, 149 women were recaptured; one was lost to follow-up. Among the study participants, 91 (61%) women were identified on the first attempt using the name-based DHIS2 application and 126 (84%) women were identified on the first attempt using the palm-based biometric solution (Table 1). Seven percent of respondents required three or more attempts for re-identification in the name-based DHIS2 application, while in the palm-based biometric solution 5% (CI: 1.9–9.4) required three or more attempts for re-identification. One woman (0.6%) out of the 149 women recaptured could not be identified in the name-based DHIS2 application; all 149 women were successfully identified with the palm-based biometric application.

**Table 1.** Number of attempts by data collectors to re-identify women using name-based DHIS2 application and palm-based biometric solution

Search attempts required	Name-based DHIS2		Palm-based biometric	
	n (%)	95% CI	n (%)	95% CI
1 attempt	91 (61.1)	52.7–68.9	126 (84.0)	77.7–89.9
2 attempts	46 (30.9)	23.6–38.9	16 (10.7)	6.2–16.8
≥ 3 attempts	11 (7.4)	3.7–12.8	6 (4.7)	1.9–9.4
Not identified	1 (0.6)	0.2–3.6	0	-

The perception of the amount of time needed for re-identification is shown in Table 2. Sixty-two percent of women were identified within 15 seconds of using the name-based DHIS2 database compared with 84% of women for the palm-based biometric systems. This almost corresponded to the number of search attempts needed for identification.

**Table 2.** Data collector perception of the time needed to re-identify women registered in name-based DHIS2 database or palm-based biometric system

Variables	Name-based database		Palm-based biometric system	
	n (%)	95% CI	n (%)	95% CI
<15 sec	92 (61.7%)	53.4–69.6	126 (84.0%)	77.7–89.9
15–60 sec	45 (30.2%)	22.9–38.2	16 (10.7%)	62.6–16.8
>1 min	11 (7.3%)	3.7–12.8	7 (4.7%)	1.9–9.4
Not identified	1 (0.6%)	0.2–3.6	0	-

The palm-based biometric solution required significantly fewer attempts to successfully identify women than the name-based DHIS2 database (mean 1.2 vs. 1.5, Z = −3.822, *P* < 0.001) (Table 3). Among the 149 women recaptured for follow-up, only 16 women required more search attempts to correctly identify women with the palm-based search than the name-based search. For 47 of the women, more search attempts were needed to correctly identify them with the name-based search than the palm-based search. Finally, 86 participants were identified with the same number of attempts in both systems (Table 3). Among the 86 women who required the same number of attempts in both systems, 79 were identified at the first attempt and 7 were identified during the second attempt in both systems.

**Table 3.** Wilcoxon rank test between two independent systems for re-identification of women

Number of attempts	N = 149	Sum ranks	Z	P value (2 tailed)
Palm-based biometry>DHIS2 name-based search	16	1931.5	−3.822	<.001
Palm-based biometry < DHIS2 name-based search	47	5565.5		
Palm-based biometry = DHIS2 name-based search	86			

### Data collectors’ perceptions

In a group discussion, the data collectors reported that the palm-based biometric application was easy to use and that they were proud and excited to use it in their work. Data collectors also reported that, the desired woman’s identifiers typically were the top option on their list of potential matches in the palm-based biometric application. One data collector mentioned that:

I like Element (palm-based biometric solution) because I could easily find the record of the woman I was searching for, and it was accurate. There are fewer chances for error.

(During re-identification) the woman whose hand it was, her name (identifiers) showed up on top of the list.

During data collection, some of the study participants were initially reluctant to allow a photo of their palm to be taken. However, when the women observed that no picture was produced or stored and instead only a code was produced (i.e. Element ID), they were comfortable participating in the trial and allowed the data collectors to capture palm pictures.

I have not faced many troubles (while testing this palm-based technology). Women (study participants) understand easily when we explain it

The data collectors also compared the use of the palm-based biometric to the more commonly used fingerprint biometrics. They reported that it would be much less challenging to convince women to use palm-based biometry than fingerprint capture, which is an alternative to legal signature for illiterate persons in Bangladesh. For example:

For fingerprints, especially the left thumb, there is presumably going to be less responsiveness, because our forefathers used left thumbprints while dealing with property ownership documents.

The data collectors also indicated that they felt that the acceptance of the palm–based biometric solution would not be challenging. A data collector stated that:

… as far as Element (palm-based biometric) works, it would be acceptable, because people of Bangladesh are becoming tech-savvy. They want something new. Many people have smartphones now even in villages. Smartphones are available in every home. Even if they do not have a Smartphone, households have access to cable networks (multiple TV channels).

## Discussion

In this study, we have, for the first time, assessed the ability of a palm-based biometric system to accurately identify women of reproductive age in a field setting in a low- or middle-income country. In addition, this is the first time, to our knowledge, that a comparative evaluation has been done between a name-based searching system to a palm-based biometric identification system for accuracy and speed. We found that although data collectors were able to correctly identify the majority of women with both systems, palm-based searching was significantly more efficient than name-based searching. Out of the total 149 mothers, one woman could not be identified in the name-based DHIS2 application, while all 149 women were successfully identified with the palm-based biometric application. Although, we were not able to identify the reason behind the failed attempt with the name-based application, this is unsurprising given the multiple possible name and spelling options which could have been used.

The palm-based biometric solution was easy to use by relatively low-skilled personnel with minimal training and no additional hardware was needed beyond a standard android device. We measured subjective time needed for each identification as the exact time was not important, while the personnel’s perception of the time needed was critical to their potential future acceptance of such a tool. In reality, the perceived time was very similar to the number of attempts needed suggesting that this was a reasonable way of estimating time. Interviews with data collectors using the system did not identify any notable challenges to implementation except that taking seven images of the palm during the registration was time consuming and may cause participants to lose patience.

Provision of high-quality care requires one to be able to easily identify an individual and gather their existing records. In many countries, unique identifiers are not routinely assigned to the entire population, leaving notable gaps around those who are the hardest to reach and most in need of care. Although fingerprints have long been the gold standard biometric identifier, capturing them typically requires an external device in addition to a smart phone or computer for data collection and requires significant storage capacity to maintain each required image. The palm-based application, on the other hand, uses existing mobile devices and stores no images, thus requiring minimal digital storage capacity, while the unique identifier can never be used to back-calculate the image of the palm for false identification purposes.

Searching for individuals in a name-based system, such as the DHIS2 database, requires an exact, albeit partial, match. Although fuzzy matching does exist, it is not routinely available for all databases. In addition, even if fuzzy matching can be implemented in an efficient way, many people may only have a single name or a very common name within a community. Even with a perfect searching system, it is highly likely that multiple individuals will appear, with no clear mechanism to distinguish them if unique identifiers are not assigned and birthdates are unknown.

This study has several limitations. First, we did not attempt to assess the specificity of the system among women not registered into the system. The Element developers had previously stated that their tests suggest a false positive rate of approximately 1 per 10,000; since this study, improved models have been developed and should be re-evaluated in low resource settings. Our trial database had a limited number of individuals recorded, compared to what would be expected in a clinical system at scale. This low number limited our ability to assess false positives which could be frequent in a name-based identification system. Since we only assessed women of reproductive age, we cannot generalize the effectiveness of this identification system for the elderly, or the young. It is possible that a different type of system would be needed for those who are much older or have significant scaring or wrinkling on their hands. In addition, we did not test this application among clinical workers or unskilled community workers. Additional training may be needed for effective use among the various cadres of workers, or to ensure an appropriate workflow when multiple cadres of workers are involved. We conducted this study in rural Bangladesh to understand whether low-skilled data collectors would be capable of using either or both systems in their own local setting. Although we conducted mini FGDs with our three data collectors, it might have been better if there were four or more. Given their consistency, we anticipated that findings would not be different with additional participants.

Limitations aside, there is a scarcity of published articles with which to compare our study findings. To our knowledge this is first study conducted in Bangladesh to test a palm-based biometric solution by low skill data collector in a rural setting. This type of comparative study should be commonplace among other identification systems, settings and provider types, as the use of individual data collection within and for health information systems and unique identifiers will further expand and progress in the future We encourage researchers and health care providers in low- and middle-income countries to evaluate the efficiency and accuracy of their population searching databases in order to be prepared as a wide variety of biometric solutions become available.

As these study results suggest that the palm-based biometric system could easily and accurately identify individuals, we have included the palm-based application as a key part of the unique identification strategy in the eRegMat trial of an electronic health registry for pregnant women. Preliminary results from the field suggest that the government workers involved in the eRegMat trial prefer this identification method over the name-based alternatives. The research team will formally evaluate that implementation later. Since this study involved the observation and measurement of the health workers’ behavior, there was room for a potential Hawthorne effect, where behaviour changes due to observation. However, our methodology and final results were based on triangulation of data collected through a variety of methods preliminarily suggesting a minimal Hawthorne effect.

## Conclusions

A palm-based biometric system, from Element, Inc., offers a technology available on mobile devices to identify patients at home or at point-of-care when unique identifiers are not routinely available. The palm-based biometric system appears to be more efficient than searching based on name alone and data collectors reported that it was both accurate and easy to operate. Our findings imply that palm-based biometrics on commonly used mobile devices may be the next step in establishing unique identifiers in remote and rural settings. For future implementation in low-resource settings, it will be necessary to test this application within clinical workflows to identify operational challenges and further refine recommendations for appropriate use. Using such technology can revolutionize the way individuals are identified within a heath information system, thereby facilitating continuity of care and avert avoidable and potentially catastrophic misidentification errors.

## Supplementary Material

Supplemental Material

## Data Availability

The dataset analysed during the current study is available in the Research Administration of icddr,b (http:// www.icddrb.org/component/content/article/10003-datapolicies/1893-datapolicies).

## Paper context

In low-and middle-income countries, the lack of unique identifiers for patients poses a barrier for the efficient delivery of health services. Biometric solutions have the potential to improve patient identification accuracy in these settings. Our study compares the searching accuracy of a palm-based biometric solution with a name-based system for improved accuracy of patient identification. This paper highlights the feasibility of establishing a point-of-care biometric system on mobile devices in rural and remote settings.
