# Supplementary material for: Comparison of a palm-based biometric solution with a name-based identification system in rural Bangladesh
Source: Glob Health Action. 2022 Mar 28;15(1):2045769. doi: 10.1080/16549716.2022.2045769 (PMC8967207; doi:10.1080/16549716.2022.2045769)
Supplement: Supplemental Material [file ZGHA_A_2045769_SM2057.docx]

Supplementary File 1: Flowchart showing process of enrolment, registration and re-identification
